# Management of Mechanical Nasal Obstruction Isolated or Associated to Upper Airway Inflammatory Diseases in Real Life: Use of both Subjective and Objective Criteria

**DOI:** 10.1007/s11882-023-01104-y

**Published:** 2023-08-10

**Authors:** Carla Merma-Linares, M. Dolores Martinez, Miriam Gonzalez, Isam Alobid, Enric Figuerola, Joaquim Mullol

**Affiliations:** 1 Department of Otorhinolaryngology- Head and Neck Surgery, Hospital Universitari de Tarragona Joan XXIII, Doctor Mallafre Guasch 4, 43005 Tarragona, Catalonia Spain; 2 Institut d‘Investigació Sanitària Pere Virgili, Tarragona, Catalonia Spain; 3 Department of Otorhinolaryngology, Rhinology Unit & Smell Clinic, ENT Department, Hospital Clínic of Barcelona, Barcelona, Catalonia Spain; 4 Skull Base Unit, ENT department, Hospital Clinic of Barcelona, Barcelona, Spain; 5 Clinical & Experimental Respiratory Immunoallergy, Institute of Biomedical Research “August Pi i Sunyer” (IDIBAPS), CIBERES, Barcelona, Catalonia Spain; 6 Present Address: Universitat de Barcelona, Barcelona, Spain; 7 Present Address: Universitat Rovira i Virgili, Tarragona, Spain

**Keywords:** Diagnostic Techniques, Nasal Obstruction, Allergic Rhinitis, CRSwNP, Nasal Surgical Procedures

## Abstract

**Purpose of Review:**

Mechanical nasal obstruction (MNO) is a prevalent condition with a high impact on patient’s quality-of-life (QoL) and socio-economic burden. The aim of this study was to determine the usefulness of both subjective and objective criteria in the appropriate management of MNO, either alone or associated to upper airway inflammatory diseases such as allergic rhinitis (AR) or chronic rhinosinusitis with nasal polyps (CRSwNP).

**Recent Findings:**

A long debate persists about the usefulness of subjective and objective methods for making decisions on the management of patients with nasal obstruction. Establishing standards and ranges of symptom scales and questionnaires is essential to measure the success of an intervention and its impact on QoL. To our knowledge this is the first real-life study to describe the management of MNO using both subjective and objective criteria in MNO isolated or associated to upper airway inflammatory diseases (AR or CRSwNP).

**Summary:**

Medical treatment (intranasal corticosteroids) has a minor but significant improvement in MNO subjective outcomes (NO, NOSE, and CQ7) with no changes in loss of smell and objective outcomes. After surgery, all MNO patients reported a significant improvement in both subjective and objective outcomes, this improvement being higher in CRSwNP. We concluded that in daily clinical practice, the therapeutic recommendation for MNO should be based on both subjective and objective outcomes, nasal corrective surgery being the treatment of choice in MNO, either isolated or associated to upper airway inflammatory diseases, AR or CRSwNP.

## Introduction

Nasal obstruction (NO) is defined as the subjective perception of insufficient airflow through the nasal cavity affecting around 30–40% of the general population. It is one of the most common complaints to the general practitioner and specialists [1]. It has been associated with a significant impairment in quality of life (QOL). Its etiology could be multifactorial, including inflammatory, neurological, functional, and mechanical causes [1]. Due to septal deformity, mechanical nasal obstruction (MNO) is highly prevalent, ranging from 22% (newborns) to 90% (adults) [2].

MNO impacts on loss of smell (LoS) and correlates with objective nasal permeability [3] as well as upper airway inflammatory diseases (AR, CRS) [4, 5]. Subjective perception is an obstacle for the evaluation of NO since patients with a similar degree of NO can differently report the symptom. A variety of diagnostic tools, both subjective (VAS, symptom scores, questionnaires) and objective (i.e. intranasal examination, acoustic rhinometry (AcR)), have been used to allow a reliable and standardized quantification [6].

The diagnosis of its cause is essential for selecting the appropriate therapy and it might be complex. Pharmacological therapy (intranasal corticosteroids) is recommended when the etiology is inflammatory or functional and surgery may be needed when the medical management fails. Some studies suggest that corticosteroid nasal spray in patients with MNO is not effective and only delays the proper treatment. Since there is not enough evidence to prove that septoplasty alone is effective in improving NO [7], a combination of surgical techniques may be needed depending on the complexity of the anatomical alteration [2]. In addition, surgery for patients with severe anatomical NO can be expensive initially but is cost-effective in the long term [8].

The aim of this study was to determine the usefulness in daily clinical practice of both subjective and objective criteria in the appropriate management of MNO, either alone or associated to upper airway inflammatory diseases, AR or CRSwNP. In addition, the results of this study will help to create an algorithm to identify the underlying disease as well as to define a personalized treatment approach.

## Materials and Methods

### Study Population

**Fig. 1:**
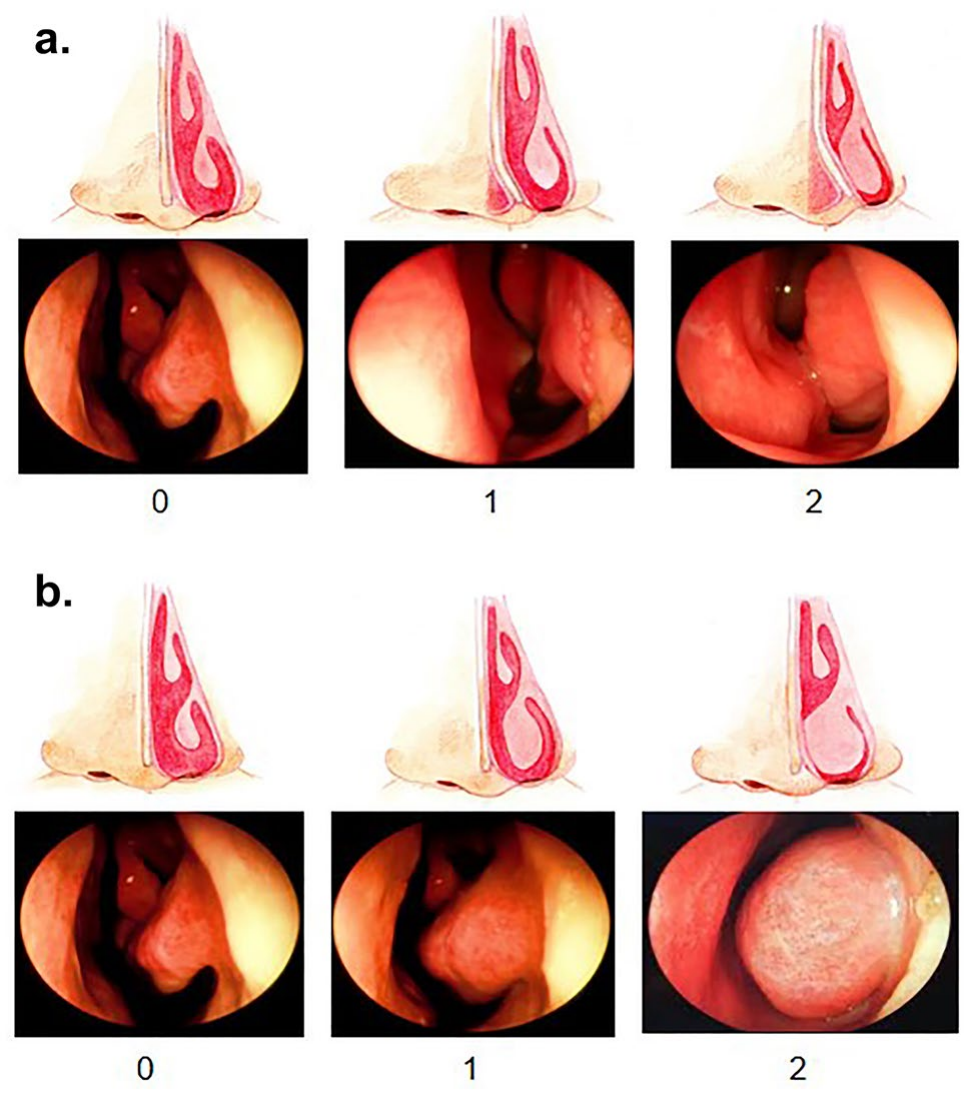
Objective criteria score of mechanical nasal obstruction (0–6). **a** Nasal septum score (0–2): 0 = “no septum deformity”; 1 = “non obstructive septum deformity”; 2 = “obstructive septum deformity”. **b** Inferior turbinate score (0–4): 0 = “no inferior turbinate enlargement”; 1 = “non obstructive turbinate enlargement; 2 = “obstructive turbinate enlargement

This is a real-life, prospective study conducted from January 2015 to December 2019. A control group without MNO and a study group with moderate-severe MNO (Likert scale ≥ 2 and objective NO score of 2 in the nasal septum or lower turbinates in nasal endoscopy, Fig. 1), referred to the Rhinology Unit, ENT Department, Hospital Universitari Joan XXIII were included. All the subjects were aged 16 years or older, able to understand the indications. Informed consent was provided by each participant.

Exclusion criteria: subjects under 16 years of age, previous nasal surgery, disorders of the nasal valve area or lateral nasal wall that could justify the NO, less than 6 months wash-out in the case of treatment with immunotherapy, psychiatric or neurocognitive impairment (lack of understanding to give answers to subjective scales), concomitant tumoral or granulomatous diseases, or not able to provide informed consent. CRSwNP patients which nasal polyps (NP) did not allow an optimal examination of the nasal structures were also excluded.

**Fig. 2:**
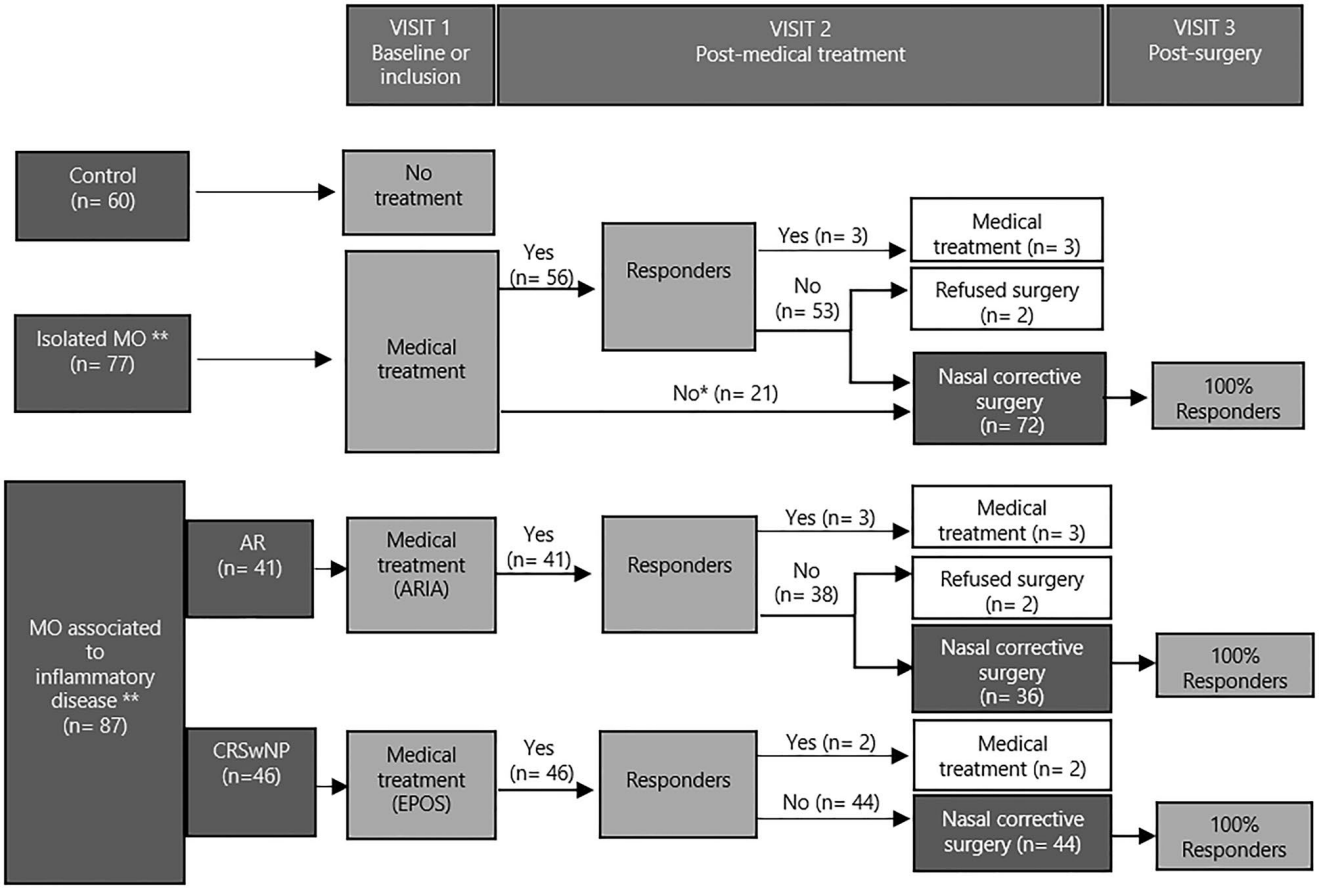
Group distribution according to treatment, follow-up and results. Abbreviations: AR, Allergic rhinitis; ARIA, Allergic Rhinitis and its impact on Asthma, CRSwNP, Chronic rhinosinusitis with nasal polyps. EPOS, European position paper on rhinosinusitis and nasal polyps. Visit 2 (post-medical treatment): at least 3 months after visit 1 (to determine the response to medical treatment). Visit 3 (post-surgery): at least 3 months after surgery to determine the response. *Patients who refused the treatment. ** None of the groups required treatment with systemic corticosteroids

Subjects were divided into two groups (Fig. 2). Group 1 (control; n = 60), male and female volunteers > 18 years old without MNO or sinonasal disease. Group 2 (MNO; n = 164) included those with isolated MNO (n = 77), and MNO associated to upper airway inflammatory disease (n = 87). AR patients (n = 41) were defined according to ARIA and GEMA guidelines (with relevant nasal and/or ocular symptoms after allergen exposure caused by IgE mediated inflammation and measured by skin prick test and/or serum specific IgE) [9, 10]. CRSwNP patients (n = 46) were defined according to EPOS [11] and POLINA [12] guidelines (chronic inflammation of the nose and the paranasal sinuses characterized by two or more symptoms, one of which should be either nasal blockage/obstruction/congestion or nasal discharge (anterior/posterior nasal drip) ± facial pain/pressure or ± reduction or loss of smell, and for > 12 weeks), and confirmed by nasal endoscopy and sinonasal CT scan.

### Study Design

The study design consisted of three visits. Visit 1 (baseline or inclusion): medical treatment was indicated (200 µg intranasal mometasone furoate/day for 12 weeks) to all patients with MNO (systemic corticosteroids were not indicated). Visit 2 (post-medical): the post-medical treatment at least 3 months after visit 1 later to determine the response to medical treatment. Visit 3 (post-surgery): at least 3 months after surgery to determine the response. Subjective scales and questionnaires (VAS for NO and LoS/NOSE/CQ7), and objective examination (nasal endoscopy and AcR) were evaluated in each visit.

The improvement criteria were assessed after the medical (visit 2) and surgical (visit 3) treatments. These were: having a NO Likert score < 2 and non-obstructive clinical examination (MNO score < 2 for all nasal structures: both lower turbinate and nasal septum). Those who did not improve after medical treatment, were referred for corrective nasal surgery. According to nasal examination* surgery included lower turbinate reduction (out fracture and volumetric tissue reduction with lower mucosa thermoplasty using bipolar forceps) and/or nasal septoplasty including functional endoscopic sinus surgery in patients with CRSwNP. *Prior to surgery (in the operating room), the clinical examination was confirmed (objective score).

### Outcomes

#### Clinical, Demographic and Anthropometric Data

Data was also collected on the duration of medical treatment and the presence of comorbidities (Table 1). The severity of AR (mild, moderate, or severe) was assessed according to modified ARIA classification [10]. Age, gender, weight, height and BMI were also recorded at visit 1.

**Table 1:** Demographic and clinical features of the study groups

Demographic and clinical features	Control (n = 60)	Isolated MO (n = 77)	MO associated to upper airway inflammatory disease (n = 87)	MNO + AR (n = 41)	MNO + CRSwNP (n = 46)
Gender, N (%)					
-Female	45 (75.0)	30 (39.0) ***	38 (43.7) ***	17 (41.5) ***	21 (45.7) ***
-Male	15 (25.0)	47 (61.0) ***	49 (56,3) ***	24 (58.5) ***	25 (54.3) ***
Age, years, mean (SD)	35.2 (12.3)	37.3 (13.6)	41.9 (15.7) **	34.2 (13.9)	48.6 (13.9) ***, ****, *****
Weight, Kg, mean (SD)	58.0 (20.3)	72.2 (14.4) ***	75.0 (14.6) ***	72.9 (15.5) ***	76.8 (13.6) ***
Height, cm, mean (SD)	165.8 (7.6)	169.1 (10.7) **	170.0 (8.6) **	170.4 (8.7) **	169,7 (8.5) **
BMI, Kg/m^2^, mean (SD)	21.1 (7.1)	25.1 (3.9) ***	25.9 (4.5) ***	25.1 (4.7) **	26.6 (4.2) ***
Comorbidities, n (%)					
- CRSwNP	0 (0)	0 (0)	46 (56.9)	0 (0)	46 (100)
- Allergic Rhinitis	0 (0)	0 (0)	53 (60.9)	41 (100)	12 (26.1)
- N-ERD	0 (0)	0 (0)	7 (8.0)	0 (0)	7 (15.2)
- Asthma	0 (0)	1 (3.9)	18 (20.7)	9 (22.0)	9 (19.6)
- OSA	0 (0)	9 (11.7)	6 (6.9)	2 (4.9)	4 (8.7)

The Chi-square test was used to perform a comparative analysis of the different phenotypes for categorical variables and ANOVA test for quali-quantitative variables (Kruskall-Wallis test if a non-parametric test was required)

*AR* allergic rhinitis, *CRSwNP* chronic rhinosinusitis with nasal polyps, *BMI* Body mass index, *MNO* Mechanical nasal obstruction, *N-ERD* NSAID-exacerbated respiratory disease, *OSA* Obstructive Sleep Apnea

*p < 0.05; **p < 0.01; *** p < 0.001, differences with respect to the control group. **** p < 0,001, difference with respect to the isolated MNO group; ***** p ≤ 0,001, difference with respect to the AR group

#### Subjective Outcomes

(A) Likert scale for NO (0, asymptomatic; 1, mild; 2, moderate; and 3, severe)
(B) VAS (from 0 cm, “no symptom”, to 10 cm, “the most severe symptom”) for NO and LoS.
(C) NOSE scale (0–100), which evaluates the severity of 5 items in the last month: nasal congestion or stuffiness, nasal blockage or obstruction, trouble breathing through the nose, trouble sleeping, and unable to get enough air through the nose during exercise or exertion. Each scored from 0 to 4 and the sum ranged from 0 to 20, which is then multiplied by 5 [7].
(D) A CQ7 questionnaire (0–28) assessed the frequency (from 0 “none of the time” to 4 “all of the time”) over the previous week of the following 7 items: nasal stuffiness/blockage/congestion, sinus pressure/facial pain, breathing through the mouth because breathing through the nose was not possible, difficulty in completely clearing the nose even after repeated blowing, ability to work/learn in school/do the things you need to do, awakening in the morning with nasal stuffiness/blockage/congestion, and sleep affected by nasal stuffiness/blockage/congestion. Higher scores indicate greater problems associated with nasal congestion [13].

#### Objective Outcomes

(A) Although objective for the patient, nasal endoscopy is a subjective method for the explorer. To avoid bias, certain criteria were followed for data collection (Fig. 1). A 5 mm flexible nasal endoscope was performed to examine the degree of NO according to the following classification: septum deformity [4] (0, no obstruction or without nasal deformity; 1, partial obstruction or non-obstructive septum deformity; or 2, total obstruction or obstructive septal deformity), and the inferior turbinate enlargement (0, no obstruction or eutrophic lower turbinate; 1, partial obstruction or non-obstructive; or 2, total obstruction or obstructive) (Fig. 1). The nasal polyp score (NPS,0–8) [14], was assessed in all CRSwNP patients considering a score of < 5 as mild-moderate and of ≥ 5 as severe [9]. A sinonasal CT scan was also carried out in all CRSwNP patients (indicated in visit 1) to evaluate the disease extension and the Lund & Mackay score (LMS, 0–24) assessed the level of sinus occupation [15] (frontal, anterior and posterior ethmoidal cells, maxillary, sphenoid sinus) with a score (0, no opacification; 1, partial opacification; or 2, complete opacification) being the ostiomeatal complex scored with 0 (not obstructed) or 2 (obstructed). Each side of the nose was scored separately (0–12) and a final LMS score provided (0–24). An aplastic (absent) frontal sinus received a score of 0.
(B) AcR (“The acoustic Executive version 3.2.0.1344 i386-win32. Spanish language licensed by *Optomic* and developed by Factree [software services]) in accordance with the Standardization Committee on AcR guidelines [16] allowed evaluation of the minimum cross-sectional area (MCA) expressed in cm^2^ and the volume of the first 5 cm of each nostril (Vol_0-5_) expressed in cm^3^. The results were described as “total values” resulting from the sum of the two nostrils. Due to a technical problem and delayed replacement of the acoustic rhinometer, not all the patients underwent AcR at visit 2, but they did after visit 3.

### Statistical Analysis

The statistical analysis was performed using STATA IC13 statistical software package. The following tests were used: a) Bartlett’s test for inequality of population to determine the homogeneity of variances. b) A descriptive analysis was carried out for the demographic characteristics. c) The chi-square test was used to make a comparative analysis between the different phenotypes for categorical variables. d) The ANOVA test to analyze the differences among group means. Previously, the normality in frequentist statistics was determined by the Shapiro Wilk test, and the non -parametric Kruskal Wallis test was used when required. e) Paired t-test to test whether or not there is a difference between two population means when the distribution is normal and Wilcoxon signed-rank when samples cannot be assumed to be normal. f) Pearson’s test to find linear correlations between quantitative variables in a normal distribution or the Spearman’s correlation test (rho) for non-parametric variables. The correlation was classified as weak (r ≤ 0.4), moderate (0.4 < r < 0.8) or strong (r > 0.8). For all statistical tests, the level of significance was set at 0.05.

## Results

### Clinical and Demographic Characteristics (Table 1)

Two hundred and twenty-four subjects were enrolled, 50.5% being female. The groups were homogeneous in terms of gender with the exception of the control group in which women predominated (75%). The mean age in the different groups was similar, except for the CRSwNP group being the oldest (48.6 years; p < 0.001).

In patients with MNO, atopy was diagnosed by a positive skin prick test in 60.9% of those with upper airway inflammatory diseases and 20.7% with bronchial asthma. Among AR patients (modified ARIA classification) [10], 69.3% presented mild, 29.3% moderate, and 2.4% severe AR. For comparison analysis among these groups, the only patient with severe rhinitis was ruled out. AR severity did not have any impact on NO subjective scores (VAS, NOSE, CQ7).

The 26.1% of the CRSwNP patients also had AR but they had similar NPS compared to the rest of the group and 15.2% had N-ERD.

### Evaluation of Outcomes at Baseline

#### Subjective Outcomes

Nasal obstruction. All MNO patients from the different groups had higher NO scores compared with the control (p < 0.001). Those with isolated MNO had similar NO values to the AR patients. Interestingly, the CRSwNP patients had a higher score of NO-VAS (8.2 ± 1.3 cm) and NOSE score (87.2 ± 2.7) with more severe nasal congestion and trouble breathing through the nose than the isolated MNO (p < 0.05). Likewise, the CRSwNP group had higher CQ7 scores in nasal obstruction/blockage/congestion and facial pressure (p < 0.05). Both, AR and CRSwNP groups, presented similar values (Table 2).

**Table 2:** Effects of medical and surgical treatment on the objective and subjective variables of Nasal Obstruction and loss of smell in the different MNO phenotypes

Subjective and objective variables		Isolated MNO (n = 77)	MNO associated to upper airway inflammatory disease (n = 87)		
				MNO + AR (n = 41)	MNO + CRSwNP (n = 46)
Nasal Obstruction, VAS^a^ (0-10 cm), mean (SD)	B	7.8 (0.8)	8.1 (1.1) *******	8.0 (0.7)	8.2 (1.3) *******
	M	7.2 (1.2)	7.3 (1.3)	7.2 (1.0)	7.3 (1.5)
	S	2.0 (1.5)	2.1 (1.3)	2.4 (1.5)	1.8 (1.1)
	Δ_B-M_	-0.7 (0.8) *	-0.7 (1.2) **	-0.7 (0.9) **	-0.8 (1.4) *
	Δ_B-S_	-5.8 (1.6) **	-6.0 (1.8) **	-5.5 (1.7) **	-6.4 (1.8) **, ****
	Δ_M-S_	-5.3 (2.0) ***	-5.3 (1.9) ***	-4.8 (1.7) ***	-5.7 (1.9) ***, ****
Loss of smell, VAS^a^ (0-10 cm), mean (SD)	B	3.5 (2.9)	5.7 (3.1) *******	4.1 (2.7)	7.2 (2.7) *****, *******
	M	3.5 (3.1)	5.3 (3.0) ********	3.8 (2.7)	6.6 (2.7) *********, *******◊◊◊, ##
	S	1.6 (1.8)	3.1 (2.5) ********	2.3 (2.3)	3.8 (2.5) *****, *******
	Δ_B-M_	-0.2 (1.4)	-0.3 (0.7)	- 0.3 (0.4)	-0.4 (0.8)
	Δ_B-S_	-2.1 (2.9) **	-2.7 (2.6) **	-1.9 (1.9) **	- 3.4 (2.9) **, ****, ******
	Δ_M-S_	-2.0 (2.9) ***	-2.4 (2.5) ***	-1.7 (1.9) ***	-2.9 (2.8) ***
NOSE^b^, scale (0–100), mean (SD)	B	82.7 (10.1)	87.3 (11.6) *******	85.6 (11.0)	88.8 (12.0) *******
	M	75.1 (16.0)	79.3 (15.3)	77.2 (13.5)	81.0 (16.6)
	S	16.7 (12.5)	20.0 (14.9)	23.9 (15.7) ******	16.7 (13.5) ****
	Δ_B-M_	-7.9 (11.2) *	-7.8 (9.5) *	-8.3 (9.7) *	-7.4 (9.3) *
	Δ_B-S_	-66.8 (16.2) **	-67.7 (19.2) **	-62.0 (20.3) **	-72.5 (17.0) **, ****
	Δ_M-S_	-60.1 (18.6) ***	-61.2 (20.9) ***	-54.8 (20.4) ***	-66.3 (20.0) ***
CQ7^c^, scale (0–28), mean (SD)	B	20.1 (3.6)	21.4 (3.3) *******	21.2 (3.0)	21.5 (3.6) ******
	M	18.2 (4.1)	18.2 (4.1) ******	19.3 (3.8)	20.1 (4.6)
	S	3.6 (2.9)	4.8 (3.8) ******	5.7 (3.9) *******	4.0 (3.5)
	Δ_B-M_	-1.7 (2.4) **	-1.6 (2.4) *	-2.0 (2.7) *	-1.3 (2.2)
	Δ_B-S_	-16.7 (4.4) **	-16.8(4.3) **	-15.6 (4.1) **	-17.7 (4.3) **, ****
	Δ_M-S_	-15.1 (4.6) ***	-15.5 (4.8) ***	-14.1 (4.2) ***	-16.6 (5.1) ***, *****
Nasal endoscopy score (0–6), mean (SD)	B	5.0 (0.8)	5.0 (0.7)	5.0 (0.9)	5.0 (0.6)
Total MCA, cm^2^, mean (SD)		1.0 (0.3)	0.9 (0.3)	0.9 (0.3)	1.0 (0.3)
		1.0 (0.4)	1.0 (0.3)	0.9 (0.3)	1.0 (0.3)
		1.1 (0.2)	1.2 (0.3)	1.1 (0.3)	1.2 (0.3)
		0.1 (0.3)	0.0 (0.2)	0.1 (0.2)	0.0 (0.2)
		0.2 (0.3)	0.2 (0.3) **	0.2 (0.4) *	0.2 (0.3) **
		0.1 (0.1)	0.2 (0.1) ***	0.2 (0.1)	0.2 (0.1) ***
Total Vol_0-5_, cm^3^, mean (SD)	B	9.9 (2.9)	9,8 (2.9)	9.7 (2.8)	9.9 (2.9)
	M	10.1 (3.0)	9,8 (2.5)	10.5 (2.6)	8.9 (2.2)
	S	11.3 (2.3)	11,8 (3.2)	11.6 (3.0)	12.0 (3.3)
	Δ_B-M_	+0.6 (2.3)	-0.3 (1.7) ******	+0.2 (1.3)	-1.1 (1.9) ******
	Δ_B-S_	+1.5 (3.3)	+2.1 (3.1) **	+2.0 (3.4) *	+2.3 (2.9) **
	Δ_M-S_	+1.0 (3.1)	+1.8 (0.5) ***	+1.2 (3.4)	+2.5 (2.6) ***

ANOVA test for comparison between groups (Kruskall-Wallis test if a non-parametric test was required) and paired t-test to test whether or not there is a difference between two population means when the distribution is normal and Wilcoxon signed-rank when samples cannot be assumed to be normal (response to treatment). The delta (Δ) negative values (-) show improvement after treatment

*AR* allergic rhinitis, *CQ7* Congestion Quantifier Questionnaire 7, *CRSwNP* chronic rhinosinusitis with nasal polyps, *MCA* Minimal cross area, *MNO* Mechanical nasal obstruction, *NOSE* Nasal obstruction and septoplasty effectiveness, *SD* Standard deviation, *VAS* Visual analogue scale, *Vol0-5* nasal volumes from 0 to 5 cm, *B* Baseline scores, *M* Post-medical treatment scores, *S* Post-surgical treatment scores, *Δ B-M* Differences between post-medical and baseline scores, *Δ M-S* Differences between post-medical and post-surgical scores, *Δ B-S* Differences between baseline and post-surgical scores

*p < 0.01; **p < 0.001; difference from baseline results. ***p < 0.001; difference between medical and surgical treatment^.^ ****p < 0.05; *****p < 0.01; difference from AR. ******p < 0.05; *******p < 0.01; ********p < 0.001; difference compared to isolated MNO group

LoS was significantly higher in CRSwNP compared to all other groups (p < 0.01).

#### Objective Outcomes

NO: There were differences in the objective scores between the control and MNO group (1.1 vs 5.0; p < 0.001). Inferior turbinate enlargement was higher in those associated with upper airway inflammatory diseases but there were no differences compared to MNO alone.

Regarding AcR, both isolated MNO and AR subjects had a total MCA (1.0 ± 0.3 cm^2^ and 0.9 ± 0.3 cm^2^; respectively) and a total Vol_0-5_ (9.9 ± 0.3 cm^3^, and 9.7 ± 2.8 cm^3^; respectively) lower than the control (p < 0.01). In CRSwNP, only total Vol_0-5_ (9.9 ± 2.9 cm^3^) was lower compared to control (p < 0.001). There were no differences among MNO subjects.

NPS: Most of CRSwNP patients (56.5%) presented severe disease but NPS did not correlate with the NO subjective score or LMS. NPS correlated weakly with LoS (Rho = 0.309; p < 0.05).

Sinonasal occupation: LMS did not correlate either with NO subjective variables (VAS/NOSE/CQ7) or with LoS.

### Effect of Medical Treatment on MNO

Only 56 out of 77 subjects with isolated MNO accepted being treated with intranasal corticosteroid (Fig. 2), being the patient’s compliance of 6.5 ± 5.4 weeks. Most patients who stopped treatment earlier reported a lack of improvement. The adherence was evaluated by the time of use of the medical treatment. Patients with MNO and upper airway inflammatory diseases showed higher adherence (AR [11.5 ± 1.6 weeks] and CRSwNP [10.3 ± 2.7 weeks]) than the group with isolated MNO (p < 0.001).

#### Subjective Outcomes

In general, medical treatment achieved a minor but significant improvement (p < 0.05) in the NO subjective outcomes (VAS/NOSE/CQ7) except in CRSwNP (VAS) with no changes in LoS (Table 2).

#### Objective Outcomes

No changes were observed in clinical examination after medical treatment in any group except in severe CRSwNP in which NPS improved only in severely ill patients compared to baseline (Δ -0.5 ± 0.7; p < 0.05). Almost 50% of the patients had post-medical AcR assessment but no significant improvement was observed (Table 2).

A clinical improvement on NO (Likert < 2) after medical treatment was observed in 5.4% of isolated MNO, 7.3% in AR, and 4.3% in CRSwNP. Surgery was proposed to those who did not improve after medical treatment being performed in 93.5% of patients with isolated MNO, 87.8% with AR, and 95.6% with CRSwNP (Fig. 2).

### Effects of Surgery on MNO

#### Subjective Outcomes

After surgery, all patients with MNO reported significant improvement in all subjective outcomes (NO/LoS/NOSE/CQ7). The most significant improvement was observed in LoS from CRSwNP patients (Table 2).

Patients with isolated MNO showed a significant improvement in subjective NO (VAS/NOSE/CQ7) and LoS compared to medical treatment (Table 2).

Both AR and CRSwNP patients showed improvement in NO and LoS subjective scores after surgery with respect to medical treatment. The improvement in CRSwNP patients was higher than in AR patients despite having higher baseline scores (Table 2).

#### Objective Outcomes

Objective outcomes improved in all patients with MNO (NO score < 1 in all the structures), additionally the NPS improved significantly in CRSwNP patients (p < 0.001).

After surgery, those patients with MNO alone did not show any significant change in AcR outcomes compared to either baseline or medical treatment. AR patients showed a minor but significant improvement in both MCA and Vol_0-5_ only compared to baseline while CRSwNP patients showed significant differences with respect to both baseline and medical treatment (p < 0.05).

### Correlation Analysis

Subjective NO outcomes (VAS/NOSE/CQ7) showed a high correlation with each other and with objective outcomes in all the study population groups (Table 3). Likewise, LoS (VAS) correlated with subjective and objective NO outcomes, especially in CRSwNP, showing that a higher NO degree (NOSE and CQ7) was highly correlated with a worse LoS (p < 0.05). CRSwNP was the only phenotype in which a subjective outcome (NO-VAS) correlated with total Vol_0-5_ from AcR (Rho -0,409; p < 0.01).

**Table 3:** Correlations between subjective and objective variables of nasal obstruction and sense of smell in the different MNO phenotypes

Correlation among variables			Study population (N = 224)	Isolated MNO (n = 77)	MNO associated to upper airway inflammatory disease (n = 87)	MNO + AR (n = 41)	MNO + CRSwNP (n = 46)
	VAS for NO^b^, Rho^p^	B	0.745***	0.509***	0.02	-0.021	0.048
Likert^a^ for NO vs	NOSE^c^, Rho^p^	B	0.719 ***	0.308 **	0.146	0.095	0.185
	CQ7 ^d^, Rho^p^	B	0.681***	0.153	0.096	0.044	0.106
	NOSE^c^, Rho^p^	B	0.767***	0.198	0.355***	0.294 *	0.499***
		Δ_B-M_	0.679***	0.784 ***	0.474 ***	0.595 ***	0.381*
		Δ_B-S_	0.851***	0.561 ***	0,674 ***	0.610 ***	0.698 ***
VAS^b^ for NO vs		Δ_M-S_	0.882***	0.545 ***	0.711 ***	0.630 ***	0.707***
	CQ7 ^d^, Rho^p^	B	0.777***	0.393 ***	0.307 **	0.303 *	0.440 **
		Δ_B-M_	0.567***	0.649 ***	0.398 ***	0.648 ***	0.244
		Δ_B-S_	0.811***	0.478***	0.559 ***	0.595***	0.491 ***
		Δ_M-S_	0.832***	0.420 **	0.420 ***	0.587 ***	0.489 **
	VAS^b^ for NO vs	B	0.696***	0.281*	0.290**	0.143	0.276
		Δ_B-M_	0.285***	0.191	0.134	-0.030	0.242
		Δ_B-S_	0.500***	0.185	0.168	-0.066	0.228
		Δ_M-S_	0.561***	0.241	0.170	0.099	0.169
	NOSE ^c^ vs	B	0.607***	-0.180	0.245*	0.000	0.289*
VAS^b^ for LoS, vs		Δ_B-M_	0.381***	0.162	0.345**	0.319	0.366 *
		Δ_B-S_	0.444***	-0.159	0.259*	0.018	0.302*
		Δ_M-S_	0.557***	-0.068	0.357**	0.306	0.320*
	CQ7^d^, Rho^p^	B	0.616***	0.052	0.148	0.005	0.142
		Δ_B-M_	0.344***	0.122	0.359**	0.113	0.543***
		Δ_B-S_	0.450***	-0.109	0.299**	0.032	0.318 *
		Δ_M-S_	0.558***	-0.080	0.410***	0.291	0.369*
NOSE ^c^ vs	CQ7^d^, Rho^p^	B	0.840 ***	0.568***	0.623***	0.483**	0.606***
		Δ_B-M_	0.757***	0.793***	0.673***	0.611***	0.697***
		Δ_B-S_	0.892***	0.675***	0.743***	0.708***	0.699***
		Δ_M-S_	0.932***	0.732***	0.787***	0.785***	0.749***
Nasal endoscopy score^e^ vs	Likert for NO^b^, Rho^p^	B	0,694***	0,135	0,102	0,036	0,225
	VAS^c^ for NO, Rho^p^	B	0,666***	0,119	0,115	0,222	0,038
	VAS^c^ for LoS, Rho^p^	B	0,627***	0,142	0,207*	0,432**	0,157
	NOSE^d^, Rho^p^	B	0,602***	-0,136	0,072	0,029	0,141
	CQ7^e^, Rho^p^	B	0,644***	-0,023	0,184	0,252	0,123

Prior to the statistical analysis, the Shapiro-Wilk test was used to assess normality. Then, a non-parametric Spearman correlation test was performed. Coefficient rho = 1, perfect positive correlation; 0 < rho < 1, positive correlation; rho = 0, there is no linear relationship; -1 < rho < 0, negative correlation

*AR* Allergic rhinitis, *CQ7* Congestion Cuantifier Questionnaire 7, *CRSwNP* Chronic rhinosinusitis with nasal polyps, *LoS* Loss of smell, *MNO* mechanical nasal obstruction, *NOSE* Nasal Obstruction and septoplasty Effectiveness, *NO* nasal obstruction, *VAS* Visual analogue scale, *B* Baseline scores, *Δ *_*B-M*_ Difference in the post-medical treatment scores with respect to baseline score, *Δ *_*M-S*_ Difference in the post-medical treatment scores with respect to postsurgical score, *Δ *_*B-S*_ Difference in the post-surgical treatment scores with respect to the baseline score

^a^Range from 0 (without NO) to 3 (severe)

^b^Range from 0 (without symptomatology) to 10 cm (more symptomatology)

^c^Range from 0 (not a problem) to 100 (severe problem)

^d^Range from 0 (none of the time) to 28 (all of the time)

^e^Range from 0 (no obstruction) to 2 (nasal obstruction) for each structure (nasal septum and lower turbinates)

^p “^p” value significance * p < 0.05; ** p < 0.01; *** p < 0.001; differences among correlation variables

## Discussion

To our knowledge this is the first real-life study to describe the management of MNO using both subjective and objective criteria in MNO isolated or associated to upper airway inflammatory diseases (AR or CRSwNP). The main findings of this study were: 1st) Medical treatment by intranasal corticosteroids achieved a minor improvement in subjective NO outcomes in all subjects while in CRSwNP the response to medical treatment was higher potentially due to the inflammatory component. 2nd) Nasal corrective surgery is the treatment of choice in MNO, both isolated and associated to upper airway inflammatory diseases (AR and CRSwNP). 3rd) LoS is more frequent in MNO with upper airway inflammatory disease, particularly in CRSwNP with clear improvement after surgery. 4th) Both subjective and objective criteria are needed to assess and optimal management of MNO since both subjective (NO/LoS/NOSE/CQ7) and objective (endoscopy score) outcomes showed strong positive correlations at baseline and after treatment. And 5th) AcR did not correlate with subjective outcomes or clinical examination, except in CRSwNP patients.

A long debate persists about the usefulness of subjective and objective methods for making decisions on the management of patients with NO. Establishing standards and ranges of symptom scales and questionnaires is essential to measure the success of an intervention and its impact on QOL [5, 6, 17, 18•].

This study shows that at baseline, subjective NO in all MNO groups was higher than previously reported, especially in CRSwNP [17, 19]. This finding might be due to the moderate-severe NO (Likert) used as inclusion criteria.

After medical treatment, there was a slight clinical NO improvement in patients with MNO, with or without upper airway inflammatory disease, in accordance with other authors [8, 20]. These data suggest that intranasal corticosteroids have a minor effect on MNO [21••] and potentially may delay the surgical treatment [8].

In our study, surgery was the optimal choice showing an improvement not only in subjective but also in objective outcomes, fulfilling success criteria previously reported [17], with a change in NO-VAS ≥ 3 and a clear reduction of NOSE (≥ 30). These changes were observed to be higher in CRSwNP which can be justified by both the correction of MNO and the resection of NP. This is in accordance with previous works [22] where symptoms assessed with CQ7 such as facial pressure, nasal congestion, and headache improved significantly after surgery. Other studies have reported that surgery increases the costs at short-term but have proven to be cost-effective at long term and more successful than non-surgical management of MNO in adults [8, 20].

Concerning to LoS, this symptom was present at baseline in all the MNO groups, predominantly in CRSwNP patients. LoS is one of the major symptoms for the clinical diagnosis of CRSwNP in both the American and the European rhinosinusitis guidelines (EPOS 2020 – IRCAR2021) [23, 24••] and also considered as a clinical marker of severity [25, 26]. Other authors [23, 27] have also correlated LoS with the degree of nasal congestion being medical treatment (i.e. intranasal corticosteroids and short course of systemic corticosteroids) clearly recommended in olfactory dysfunction secondary to CRS. In this study, LoS improved only after surgery in all subjects but more significantly in CRSwNP, which supports the impact of both inflammatory and mechanical factors in this symptom [28, 29]. Our findings suggest that improvement in smell may be related to improved conduction of odorants to the olfactory neuroepithelium.

A main finding of this study was also the high positive correlation between all subjective scales (VAS/NOSE/CQ7) at baseline, medical and surgical treatments. According to some authors [5, 18•], these scoring systems are useful because they are capable of determining subjective changes (follow-up) in the assessment of treatment effects (1B: strong recommendation, moderate quality of evidence). Similarly, these scales correlated with some objective outcomes. However, the NO subjective outcomes did not correlate with NPS in CRSwNP subjects, as reported in other studies [30–32] but in contrast with others [33]. Furthermore, a small correlation was found between NPS and CT findings (LMS) in agreement with some authors [34] but in disagreement with others who reported some correlation [31]. Some studies have reported an uncertain correlation between AcR and NO subjective score systems [5, 17]. In our study, only some correlation between NO-VAS and Vol_0-5_ was found in CRSwNP patients.

**Fig. 3:**
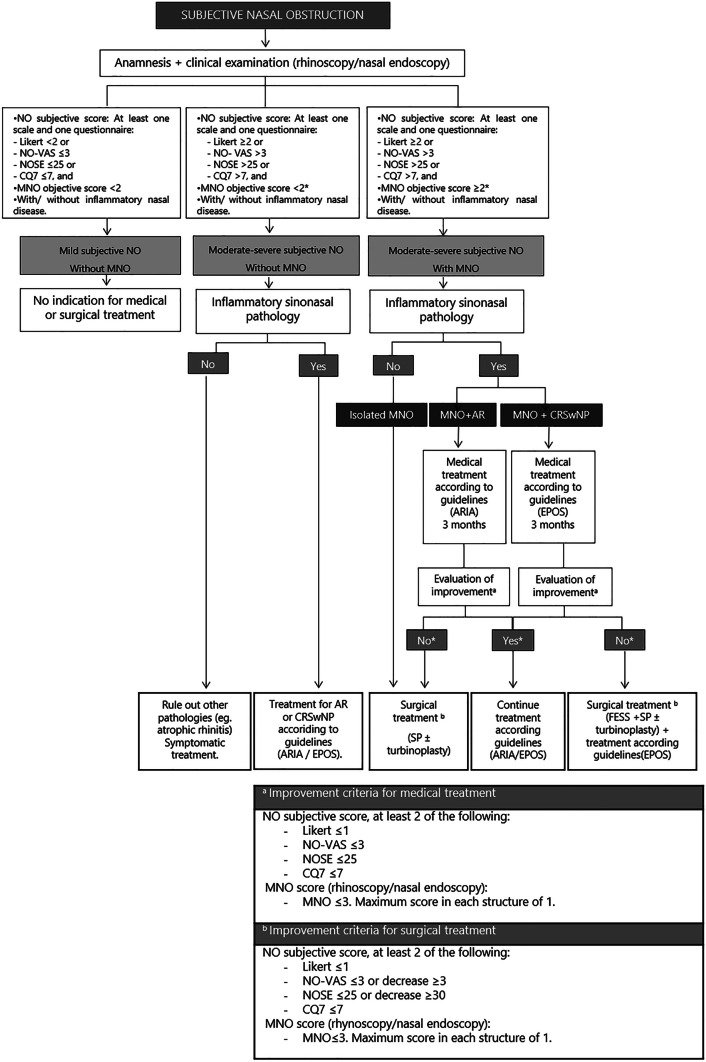
Mechanical nasal obstruction (MNO) management algorithm. Abbreviations: AR, Allergic rhinitis; CRSwNP, Chronic rhinosinusitis with nasal polyps; CQ7, Congestion Quantifier Questionnaire 7; FESS, Functional endoscopy sinus surgery; MNO, mechanical nasal obstruction; NO, Nasal obstruction; NOSE, Nasal Obstruction and Septoplasty Effectiveness; SP, septoplasty; VAS, Visual Analogue Scale. * If the score is 2, it must correspond to one of the 3 structures studied (obstructive lower left and/or right turbinate enlargement and/or septal deformity)

Finally, our study has allowed us to design an algorithm to guide the most appropriate MNO management (diagnosis and treatment) in daily clinical practice (Fig. 3).

### Strengths and Limitations

This real-life study is, to our knowledge, the first to demonstrate the effect of medical and surgical treatments in MNO alone or associated to upper airway inflammatory diseases, either AR or CRSwNP. It also proves that nasal symptoms, measured by VAS, NOSE and CQ7, highly correlated with each other and with objective outcomes, easy to use in clinical practice.

Our own MNO score by clinical examination with nasal endoscopy facilitated the data collection, being practical and easy to interpret. However, this classification will require further validation.

Regarding objective methods, AcR (easier and faster to perform) is the only objective test available at our hospital. This is the reason why methods such as anterior rhinomanometry was not used for the study. Trying to avoid bias, one investigator alone confirmed and interpreted the data.

As a main limitation, the assessment of the patients was based on the subjective perception derived from clinical anamnesis and examination by different researches from the same team, which can lead to some differences in data collection. ENTs who performed the nasal endoscopy were not blinded to the procedures performed to the patients or the rest of scales since the study was done in daily clinical practice. Physical examination was performed with rhinoscopy and nasal endoscopy following the same criteria evaluation with specific features to evaluate nasal septum and inferior turbinate and modified Lildholdt score for patients with CRS with NP to avoid bias in terms of interpretation. This MNO score by clinical examination with nasal endoscopy facilitated the data collection, being practical and easy to interpret. However, this classification will require further validation.

Likewise, surgery was not carried by a single surgeon but by the rhinology surgical team using the same standardized technique.

In addition, although QOL questionnaires were not used, NOSE and CQ7 scales reflect symptoms that would influence QOL.

### Conclusions

In daily clinical practice, medical treatment by intranasal corticosteroids is not useful in MNO alone but it may help when associated to comorbid upper airway inflammatory disease. Nasal corrective surgery is the treatment of choice in MNO, both isolated and associated to upper airway inflammatory diseases, either AR or CRSwNP. The therapeutic indication for MNO should be based on both subjective and objective outcomes while AcR, an objective assessment, may be useful but as complementary examination.

## Data Availability

The data that support the findings of this study are available for this journal.
